# Mapping motion of antiferromagnetic interfacial uncompensated magnetic moment in exchange-biased bilayers

**DOI:** 10.1038/srep09183

**Published:** 2015-03-17

**Authors:** X. Zhou, L. Ma, Z. Shi, W. J. Fan, R. F. L. Evans, Jian-Guo Zheng, R. W. Chantrell, S. Mangin, H. W. Zhang, S. M. Zhou

**Affiliations:** 1 Shanghai Key Laboratory of Special Artificial Microstructure Materials and Technology and Pohl Institute of Solid State Physics and School of Physics Science and Engineering, Tongji University, Shanghai 200092, China; 2 Department of Physics, University of York, York YO10 5DD, United Kingdom; 3 The Laboratory for Electron and X-ray Instrumentation, Calit2, University of California, Irvine, CA 92697-2800, USA; 4 Institut Jean Lamour, UMR CNRS 7198, Université de Lorraine- boulevard des aiguillettes, BP 70239, Vandoeuvre cedex F-54506, France; 5 State Key Laboratory of Electronic Thin Films and Integrated Devices, University of Electronic Science and Technology of China, Chengdu 610054, China

## Abstract

In this work, disordered-IrMn_3_/insulating-Y_3_Fe_5_O_12_ exchange-biased bilayers are studied. The behavior of the net magnetic moment Δ*m_AFM_* in the antiferromagnet is directly probed by anomalous and planar Hall effects, and anisotropic magnetoresistance. The Δ*m_AFM_* is proved to come from the interfacial uncompensated magnetic moment. We demonstrate that the exchange bias and rotational hysteresis loss are induced by partial rotation and irreversible switching of the Δ*m_AFM_*. In the athermal training effect, the state of the Δ*m_AFM_* cannot be recovered after one cycle of hysteresis loop. This work highlights the fundamental role of the Δ*m_AFM_* in the exchange bias and facilitates the manipulation of antiferromagnetic spintronic devices.

Exchange bias (EB) in ferromagnetic (FM)/antiferromagnetic (AFM) systems has attracted much attention because of its intriguing physics and technological importance in spin valve based magnetic devices^1^,^2^,^3^,^4^,^5^,^6^,^7^. After the FM/AFM bilayers are cooled under an external magnetic field *H_FC_* from high temperatures to below the Néel temperature of the AFM layers, hysteresis loops are simultaneously shifted and broadened^8^. FM/AFM bilayers are now commonly integrated in spintronic devices^9^. Nevertheless manipulation and characterization of the AFM spins are important to understand and control the EB phenomena^10^.

Rotatable and frozen AFM spins are often thought to be responsible for the coercivity enhancement and shift of the magnetization hysteresis loops^5^,^11^,^12^,^13^,^14^,^15^. Ohldag *et al.* found that a nonzero AFM net magnetic moment Δ*m_AFM_* is necessary to establish EB^16^. However, Wu *et al.* thought that EB can be established without Δ*m_AFM_*^12^. Therefore, the behavior of AFM spins is still under debate. Moreover, for FM/AFM bilayers, the rotational hysteresis loss at external magnetic field *H* larger than the saturation field is ascribed to the irreversible switching of AFM spins during clock wise (CW) and counter clock wise (CCW) rotations^11^,^17^,^18^. The EB training effect is attributed to the relaxation of the Δ*m_AFM_* towards the equilibrium state during consecutive hysteresis loops^19^,^20^,^21^,^22^,^23^,^24^. There is still a lack of direct experimental evidence.

In most studies, the information of AFM spins is *indirectly* explored from the hysteresis loops of the FM layers by micromagnetic simulations and Monte Carlo calculations^15^,^17^,^21^,^23^. Very few methods can be implemented to directly probe the AFM spins due to almost zero net magnetic moment of the AFM layers. X-ray magnetic circular dichroism and x-ray magnetic linear dichroism can detect FM and AFM spins due to their element-specific advantage^12^,^16^,^25^. In a pioneer work, tunneling anisotropic magnetoresistance (TAMR) effect has very recently been used to probe the motion of the AFM spins in AFM spintronic devices^26^,^27^. Since the TAMR depends on the orientation of the AFM spins in a *complex* way, however, the orientation of the AFM spins cannot be determined directly and in particular the issue whether the Δ*m_AFM_* exists or not is still unsolved^28^. In this work, we demonstrate clear evidence of the existence of Δ*m_AFM_* and reveal its role in EB, the training effect, and the rotational hysteresis loss for disordered-IrMn_3_( = IrMn)/Y_3_Fe_5_O_12_ ( = YIG) bilayers using anomalous Hall effect (AHE), planar Hall effect (PHE), and anisotropic magnetoresistance (AMR) measurements. Here, the YIG *insulator* is used as the FM layer such that all magnetotransport properties are contributed by the metallic IrMn layer. Galvanomagnetic measurements probe the entire IrMn layer, not only the interface as in the reported TAMR measurements^26^,^27^. The Δ*m_AFM_* in metallic IrMn is proven experimentally to arise from the interfacial uncompensated magnetic moment. It is clearly demonstrated in experiments that the EB and related phenomena are intrinsically linked to the partial pinning and irreversible motion of the Δ*m_AFM_*.

## Results

**Figure 1:**
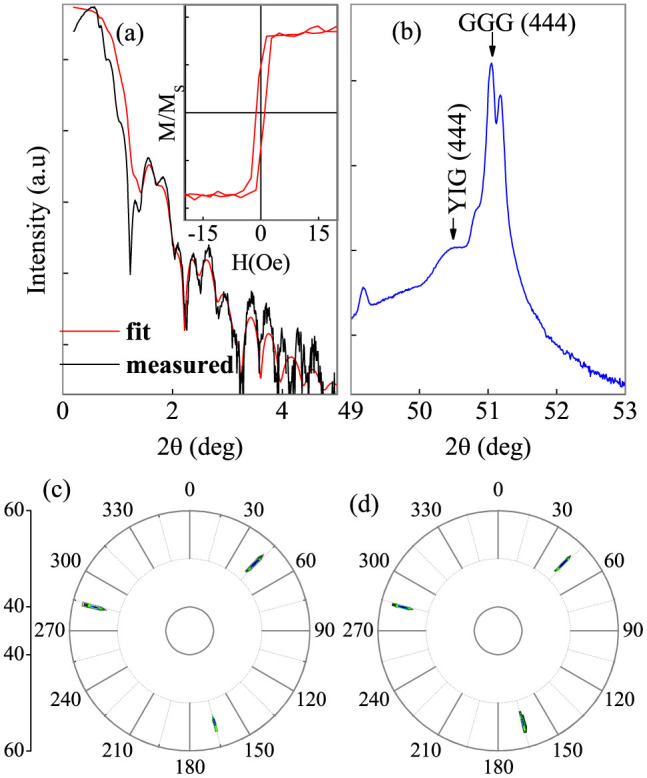
(a) Small angle x-ray reflection, (b) large angle x-ray diffraction for IrMn (5 nm)/YIG (20 nm) bilayer, Φ and Ψ scan with fixed 2*θ* for the (008) reflection of GGG substrate (c) and YIG film (d). The room temperature in-plane magnetization hysteresis loop of the YIG layer is shown in the inset of (a).

X-ray reflectivity (XRR) spectrum in Fig. 1(a) shows that YIG and IrMn layers are 20 ± 0.6 and 5.0 ± 0.5 nm thick, respectively. The x-ray diffraction (XRD) spectrum in Fig. 1(b) shows that the Gd_3_Ga_5_O_12_ (GGG) substrate and YIG film are of (444) and (888) orientations. The pole figures in Figs. 1(c) and 1(d) confirm the epitaxial growth of the YIG film. The epitaxial YIG is magnetically soft, as shown in inset of Fig. 1(a). The IrMn layer deposited at ambient temperature is proved to be polycrystalline by transmission electronic microscopy (TEM), as shown in Fig. S1 in supplementary materials. The IrMn layer is expected to be disordered because the disorder to order transformation in the IrMn layer often occurs at elevated substrate temperatures^29^. High resolution TEM results also indicate that despite the overlapping of the IrMn and YIG layers at the interface due to the YIG surface roughness, any other layer can be excluded.

**Figure 2:**
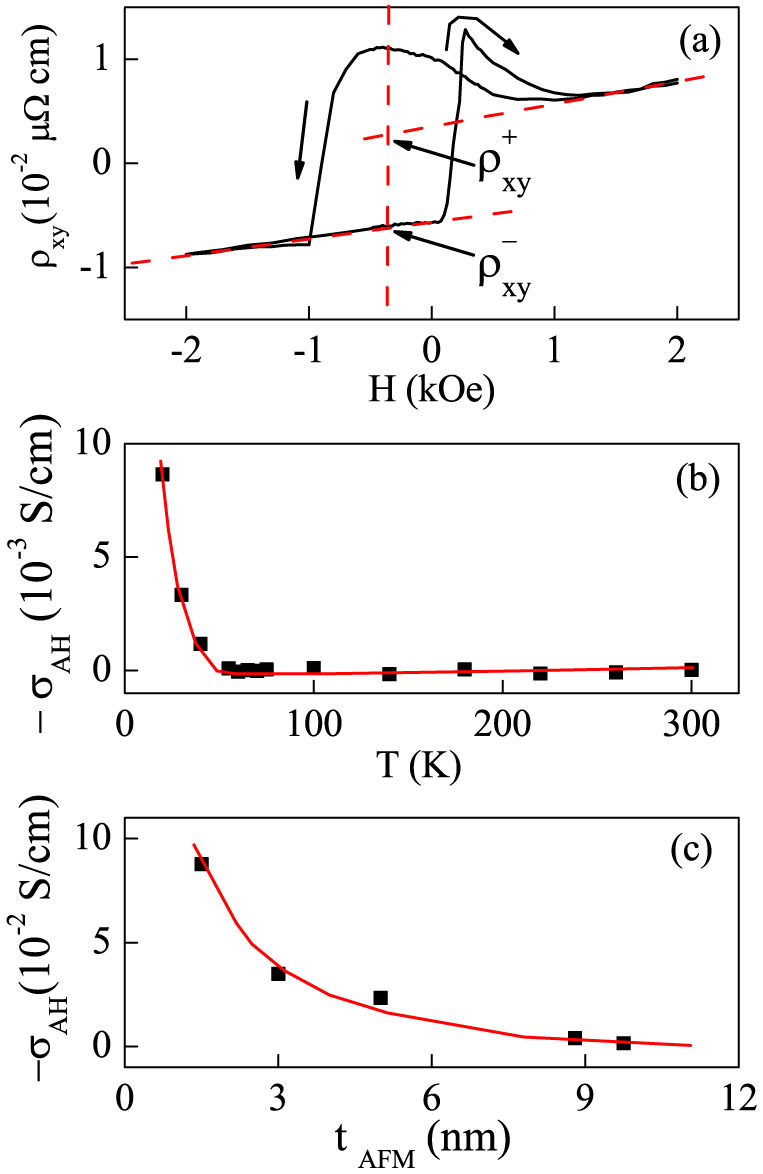
For IrMn (5 nm)/YIG (20 nm) bilayer, Hall loop at 20 K with *H* along the film normal direction (a), and AHC as a function of *T* (b). For IrMn/YIG (20 nm) bilayers, the AHC at 2 K as a function of the IrMn layer thickness (c). The solid lines in (b, c) serve a guide to the eye.

Before measurements, the films were cooled from room temperature to 5 K under *H* = 30 kOe along the film normal direction. The Hall resistivity *ρ_xy_* was measured as a function of the out-of-plane *H* at various temperatures (T), as shown in Fig. 2(a). The values of Hall resistivity at spontaneous states, 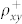 and 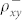, were extrapolated from the positive and negative high *H* and the anomalous Hall resistivity was obtained by the equation 
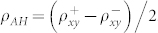
. One has the anomalous Hall conductivity (AHC) 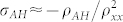 because *ρ_AH_* is two orders of magnitude smaller than *ρ_xx_*^30^. Since *ρ_AH_* decreases sharply and vanishes near *T* = 50 K, *σ_AH_* is reduced with increasing *T* and approaches zero at *T* ~ 50 K, as shown in Fig. 2(b). Interestingly, it is found that the Hall loop is shifted at low *T* and centered at zero *H* at high *T*. The AHC is consequently accompanied by the established EB.

It is essential to address the physics for the AHC in IrMn/YIG bilayers. *σ_AH_* in the present IrMn/YIG bilayers changes strongly with the IrMn layer thickness, demonstrating an interfacial nature, as shown in Fig. 2(c). The strong *T* and layer thickness dependencies of the AHC cannot be attributed to the noncollinear spin structure^31^. Otherwise, the AHC should be independent of the film thickness and change slowly with *T* due to the high Néel temperature. The strong *T* dependence also hints that the present AHC results cannot be attributed to the spin Hall magnetoresistance either^32^. As pointed above, however, the AHC is strongly related to the established EB, which is further confirmed by the vanishing AHC for the 5 nm thick IrMn films on GGG substrates in Fig. S2 in supplementary materials. As shown by the AMR results below, any FM layer at the interface can be excluded, in agreement with the TEM results. Hence, the AHC exclusively proves the existence of the IrMn interfacial uncompensated magnetic moment which is produced by the field cooling procedure.

**Figure 3:**
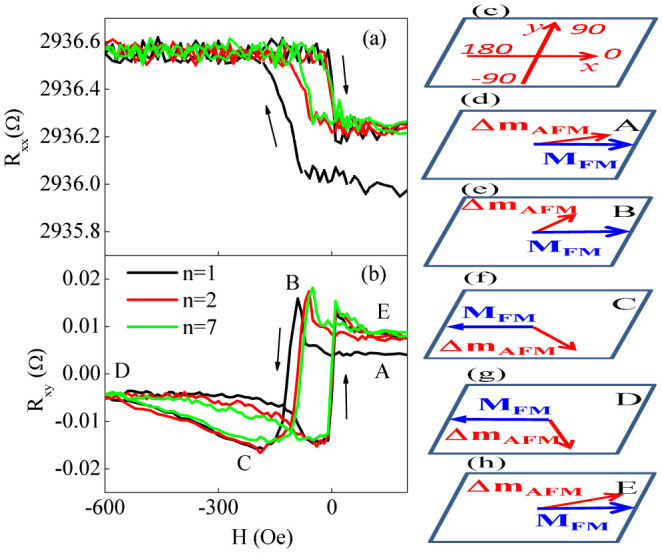
AMR curves (a) and PHE loops (b) for an IrMn (5 nm)/YIG (20 nm) bilayer for different cycles 1, 2, and 7. The film is aligned in the *x-y* plane, the sensing current *i*, *H_FC_*, and *H* are parallel to the *x* direction indicated by the schematic (c), the evolution of the orientations of Δ*m_AFM_* and FM magnetization at stages A(d), B(e), C(f), D(g), and E(h) in the descent branch of the *n* = 1 in (b).

Before measurements of the AMR curves and PHE loops, the sample was cooled from room temperature to 5 K under an in-plane *H_FC_*. Figures 3(a) and 3(b) show the AMR curves and PHE loops with consecutive cycles, where *H*, *H_FC_*, and the sensing current *i* are all parallel to the *x* axis, as shown in Fig. 3(c). Distinguished features are demonstrated in the descent branch of the first cycle, *n* = 1. Most importantly, conventional FM metallic films exhibit butterfly-shaped AMR curves at low magnetic fields and the values of the *R_xx_* at positive and negative high *H* are equal to each other^21^,^33^. In striking contrast, the IrMn/YIG bilayer displays a loop-shaped AMR curves in Fig. 3(a). The unique feature cannot be attributed to any metallic FM layer at the interface but exclusively to the interfacial uncompensated magnetic moment of the IrMn layer. Accordingly, the PHE signal and the AMR ratio are proportional to sin(2*θ_AFM_*) and 1 − cos^2^ *θ_AFM_*, respectively, where *θ_AFM_* refers to the angle between the Δ*m_AFM_* and the *x* axis^33^. More remarkably, with the monotonic change of the *R_xx_* in Fig. 3(a), one has 0 < |*θ_AFM_*(*A*)| < |*θ_AFM_*(*B*)| < |*θ_AFM_*(*C*)| < |*θ_AFM_*(*D*)| < 90° for stages A, B, C, and D^26^. In combination with the sign change of the *R_xy_* in Fig. 3(b), one has 0 < *θ_AFM_*(*A*) < *θ_AFM_*(*B*) < 90° and −90° < *θ_AFM_*(*D*) < *θ_AFM_*(*C*) < 0, as schematically shown in Figs. 3(d)–3(g). Since the IrMn layer is *far* from the negative saturation within the field of −600 Oe and the orientation of the FM magnetization *θ_FM_* with respect to the *x* axis equals 0 and 180° at positive and negative saturations, respectively, the angle between the FM and AFM magnetic moments, *θ_FM_* − *θ_AFM_*, is smaller (larger) than 90° at the positive (negative) high *H*, and the interfacial exchange coupling energy, *E_ex_* = −*J*_cos_(*θ_FM_* − *θ_AFM_*) with ferromagnetic interlayer coupling (*J* > 0), is low (high), leading to a lateral and a vertical shift in the magnetization hysteresis loop^8^,^34^,^35^,^36^. Moreover, when the *H* changes from stages B to C, the Δ*m_AFM_* is irreversibly switched from the first quadrant to the fourth one^17^. In a word, the partial rotation and irreversible switching of the Δ*m_AFM_* elucidate the intriguing physics behind the shifting and broadening of magnetization hysteresis loops^8^,^11^,^17^.

For the cycle number *n* = 1, 2, and 7, the descent branch shifts significantly whereas the ascent branch almost does not change as shown in Figs. 3(a) and 3(b), in agreement with the first kind of the EB training effect of the FM magnetization hysteresis loops in Fig. S3^19^. The athermal training effect for *n* = 1 is much larger than those of *n* > 2^20^. In particular, the PHE signal and AMR ratio at the starting stage A are smaller than those of the ending stage E, that is to say, *θ_AFM_*(*E*) > *θ_AFM_*(*A*), verifying in experiments the switching of AFM spins among easy axes in the athermal training effect^20^,^23^,^24^,^37^. The AMR curves and PHE loops in Figs. 3(a) and 3(b) also suggest that the Δ*m_AFM_* experiences different trajectories during consecutive cycles^19^,^20^,^21^,^22^.

**Figure 4:**
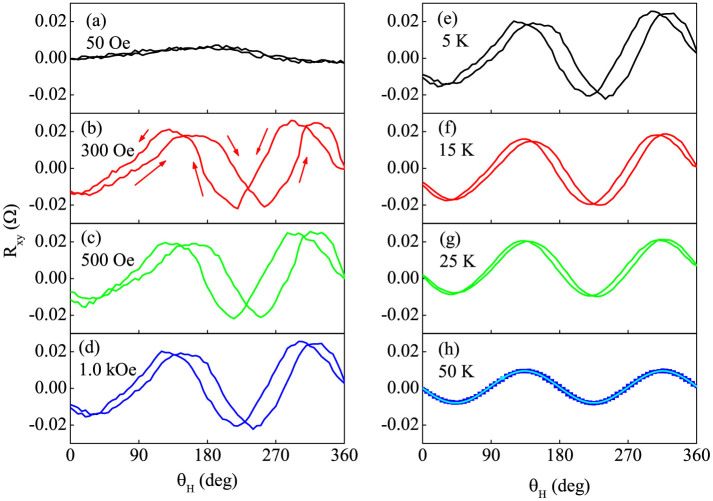
For IrMn (5 nm)/YIG (20 nm) bilayer, angular dependent PHE signal with CW and CCW senses at *H* = 50 (a), 300 (b), 500 (c), and 1000 (d) (Oe), and at *T* = 5 (e), 15 (f), 25 (g), and 50 (h) (K). *T* = 5 K in the left column and *H* = 1.0 kOe in the right column. In (h), solid cyan line refers to the *sin*(2*θ_H_*) fitted results.

Figures 4(a)–4(d) show the PHE signal as a function of *θ_H_* with CW and CCW rotations under different magnitudes of *H*. At *H* = 50 Oe, the CW and CCW curves overlap and the FM and AFM spins are expected to rotate reversibly within a small angular region. For higher *H*, the hysteretic behavior begins to occur and becomes strong for *H* = 300 and 500 (Oe). This effect starts to become weak for *H* = 1.0 kOe but still persists at *H* = 20 kOe. Figures 4(e)–4(h) show the rotational hysteresis loss under *H* = 1.0 kOe at different *T*. At *T* < 50 K, the CW and CCW curves differ from each other, indicating irreversible rotation of the Δ*m_AFM_*, and the hysteretic effect becomes weak at enhanced *T*. Near *T* = 50 K, the measured results can be fitted well with sin(2*θ_H_*) due to either the ordinary magnetoresistance effect or the spin Hall magnetoresistance^32^ and the contribution of Δ*m_AFM_* approaches vanishing, as demonstrated in Fig. S4. In a word, the hysteretic behavior of the PHE curves reproduces the rotational hysteresis loss of the FM magnetization, a fingerprint of the EB in FM/AFM bilayers^1^,^8^,^18^.

## Discussion

It is interesting to analyze the magnitude and reversal mechanism of the Δ*m_AFM_* as a function of *T*. As shown above, the galvanomagnetic effects become weak with increasing *T* and approaches vanishing at 50 K (*T_B_*). It is suggested that Δ*m_AFM_* is reduced at elevated *T* and disappears at *T_B_*. Meanwhile, the Δ*m_AFM_* is mainly switched irreversibly (reversibly) at low (high) *T*. The variation of the reversal mode with *T* confirms the validity of the thermal fluctuation model for polycrystalline AFM systems^11^,^38^. In this model, the reversal possibility is governed by the Arrhenius-Néel law and determined by the competition between the thermal energy and the energy barrier which is equal to the product of the uniaxial anisotropy and the AFM grain volume. The low *T_B_* of 50 K is likely caused by ultrathin thickness and the microstructural deterioration of the IrMn layer which is induced by the lattice mismatch between IrMn and YIG layers^26^. At *T* < *T_B_*, the energy barrier is larger than the thermal energy, leading to the irreversible process in most AFM grains. Accordingly, the EB is established and accompanied by the sizeable galvanomagnetic effects. Since more AFM grains become superparamagnetic for *T* close to *T_B_*, the Δ*m_AFM_*, galvanomagnetic effects, and the EB all approach vanishing. On the other hand, the Meiklejohn-Bean (M-B) model and the domain state model are *not* suitable for the present IrMn/YIG systems^8^,^39^. In the M-B model, AFM spins are fixed during the reversal of the FM magnetization, which is in contradiction with the present results. In the domain state model, the AFM net magnetic moment is mainly contributed by the bulk AFM^39^ whereas the Δ*m_AFM_* in the present IrMn/YIG systems mainly stems from the uncompensated magnetic moment at FM/AFM interface.

In summary, for IrMn/YIG bilayers the interfacial uncompensated magnetic moment Δ*m_AFM_* is observed by the galvanomagnetic effects. The partial rotation and irreversible switching of the Δ*m_AFM_* are directly proved to be the physical source for the exchange field, coercivity enhancement, and the rotational hysteresis loss. In the athermal training effect, the state of the Δ*m_AFM_* *cannot* be recovered after the first cycle of hysteresis loop. The present work permits a better and full understanding of EB and related phenomena in FM/AFM bilayers. It demonstrates that galvanomagnetic measurements allow to probe the behavior of the AFM layer. This technique will also shed new light on the field of AFM spintronics.

## Methods

### Sample description

A series of IrMn/YIG (20 nm) bilayers were fabricated by pulsed laser deposition (PLD) and DC magnetron sputtering on (111)-oriented, single crystalline GGG substrates. The YIG layer was epitaxially grown via PLD from a stoichiometric polycrystalline target using a KrF excimer laser. Afterwards, the IrMn layer was deposited at ambient temperature from an IrMn alloy target by magnetron sputtering, in order to avoid interfacial diffusion.

### Experimental method

Structural properties and film thickness were characterized by X-ray diffraction (XRD) and X-ray reflectivity (XRR) using a D8 Discover X-ray diffractometer with Cu K*α* radiation (wavelength of about 1.54 Å), and TEM techniques. The epitaxial growth of the YIG film was proved by pole figures with Φ and Ψ scan at 2*θ* fixed for the (008) reflection of the GGG substrate and YIG film. Cross-sectional images of the IrMn/YIG bilayers were characterized by TEM. (See supplementtal material for Hall effect of the 5 nm thick IrMn single layer film, high resolution TEM images, temperature and angular dependence of the PHE loops, and the EB training of magnetization hysteresis loops for IrMn/YIG bilayer.) Magnetization hysteresis loops of the samples were measured using physics properties of measurement system (PPMS). The magnetization (134 emu/cm^3^) of the YIG film is close to the theoretical value (131 emu/cm^3^) and the coercivity is very small, 6 Oe. The films were patterned into normal Hall bar, and the transverse Hall resistivity (*ρ_xy_*) and the longitudinal resistivity (*ρ_xx_*) were measured by PPMS.

## Author Contributions

X.Z. and S.M.Z. conceived the experiments. H.W.Z. helped with the substrates. X.Z. and L.M. fabricated the samples and carried out the measurements. J.G.Z. performed TEM specimen preparation, imaging and analysis. Z.S. has some contributions for the transport measurement setup. W.J.F., R.F.L.E., R.W.C. and S.M. contributed to manuscript preparation and the analysis and discussion for the results. X.Z. and S.M.Z. wrote the paper and all the co-authors comment on it.

## Supplementary Material

Supplementary Information Supplementary Information
