# Supplementary material for: Mapping motion of antiferromagnetic interfacial uncompensated magnetic moment in exchange-biased bilayers
Source: Sci Rep. 2015 Mar 17;5:9183. doi: 10.1038/srep09183 (PMC4361867; doi:10.1038/srep09183)
Supplement: Supplementary Information [file srep09183-s1.pdf]

Mapping motion of antiferromagnetic interfacial uncompensated magnetic moment in exchange-biased bilayers

X. Zhou,[1] L. Ma,[1] Z. Shi,[1] W. J. Fan,[1] R. F. L. Evans,[2] Jian-Guo Zheng,[3] R. W. Chantrell,[2] S. Mangin,[4] H. W. Zhang,[5] and S. M. Zhou‡ [1]

[1]*Shanghai Key Laboratory of Special Artificial Microstructure Materials and Technology and Pohl Institute of Solid State Physics and School of Physics Science and Engineering, Tongji University, Shanghai 200092, China*

[2]*Department of Physics, University of York, York YO10 5DD, United Kingdom*

[3]*The Laboratory for Electron and X-ray Instrumentation, Calit2, University of California, Irvine, CA 92697-2800, USA*

[4]*Institut Jean Lamour, UMR CNRS 7198, Universit de Lorraine- boulevard des aiguillettes, BP 70239, Vandoeuvre cedex F-54506, France*

[5] *State Key Laboratory of Electronic Thin Films and Integrated Devices, University of Electronic Science and Technology of China, Chengdu 610054, China*

‡ Correspondence author. Electronic mail: shiming@tongji.edu.cn

I. SUPPLEMENTARY MATERIAL

A. Fabrication and measurement details

A series of $IrMn_3$(=IrMn)/$Y_3Fe_5O_{12}$(=YIG) (20 nm) bilayers were fabricated by pulsed laser deposition (PLD) and DC magnetron sputtering on (111)-oriented, single crystalline $Gd_3Ga_5O_{12}$ (GGG) substrates. The base pressures of the PLD and sputtering systems were 1.0×10^{-6} Pa. The YIG layer was epitaxially grown via PLD from a stoichiometric polycrystalline target using a KrF excimer laser with the pule energy of 285 mJ. The substrate temperature was 625 °C during the deposition of the YIG layer. Then, the sample was annealed at the same temperature in an O_2 pressure of 1×10^4 Pa for 4 hours. After the sample was cooled to the ambient temperature, it was transferred without the air exposure from the PLD chamber to the sputtering chamber through a load-lock chamber. Afterwards, the IrMn layer was deposited at ambient temperature from an IrMn alloy target by magnetron sputtering, in order to avoid interfacial diffusion. The Ar pressure was 0.3 Pa during deposition of the IrMn layer. The deposition rate of IrMn was about 0.1 nm/s.

Structural properties and film thickness were characterized by X-ray diffraction (XRD) and X-ray reflectivity (XRR) using a D8 Discover X-ray diffractometer with Cu Kα radiation (wavelength of about 1.54 Å). The epitaxial growth of the YIG film was proved by pole figures with Φ and Ψ scan at 2θ fixed for the (008) reflection of the GGG substrate and YIG film. Transmission electronic microscopy (TEM) experiments were carried out in FEI/Philips CM-20 TEM with a LaB_6 filament ope rated at 200 kV at the Laboratory for Electron and X-ray Instrumentation, University of California Irvine. Cross-sectional TEM specimens were prepared in a FEI Quanta 3D FEG dual-beam system with focused ion beam (FIB). A typical FIB procedure recommended by FEI Company was used to prepare the specimens. The thin film was well protected by electron beam deposited Pt-layer before the film was exposed under the Ga-ion beam for further ion beam Pt deposition. The final thinning step using a low energy (2 kV) ion beam is crucial to minimize an amorphous layer, a damaged layer caused by Ga-ion beam, on both sides of the TEM specimens. Magnetization hysteresis loops of the samples were measured using physics properties of measurement system. The magnetization (134 emu/cm^3) of the YIG film is close to

the theoretical value (131 emu/cm^3) and the coercivity is very small, 6 Oe. The films were patterned into normal Hall bar, and the transverse Hall resistivity (ρ_{xy}) and the longitudinal resistivity (ρ_{xx}) were measured by physical property measurement system (PPMS).

B. TEM results

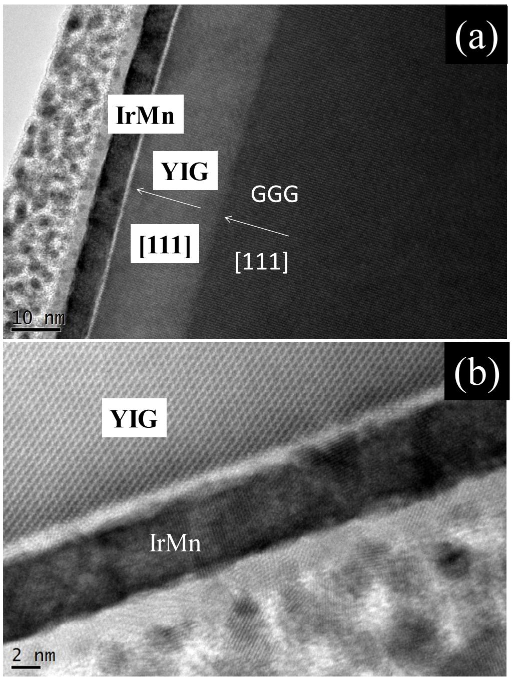

FIG. S1: In (a), cross-sectional high resolution TEM (low magnification) image of IrMn/YIG bilayers on (111) GGG substrate, where the IrMn and YIG layers are 6 nm and 20 nm, respectively. In (b), high resolution TEM (high magnification) image of the sample.

Figure S1 shows typical high resolution TEM images. The 20 nm thick YIG is grown epitaxially on the GGG (111) substrate (Fig. S1(a)) and the IrMn layer is polycrystalline (Fig. S1(b)). In Fig. S1(a), the fine Pt particles on top of the IrMn thin film form the protection layer which was deposited during TEM specimen preparation. The white area

at the IrMn/YIG interface may be produced during the preparation of the TEM sample because the milling rate at the interface is slightly higher than that in YIG. The overlapping of YIG and IrMn was observed at the interface due to the YIG surface roughness, where the root mean square surface roughness of the YIG layer is 0.35 nm.

C. Anomalous Hall conductivity of IrMn single layer films

In comparison, a 5 nm thick IrMn single layer film was deposited at ambient temperature on the GGG substrate. Figure S2 shows that the Hall resistivity versus the external magnetic field H at 10 K. The Hall resistivity is proportional to the magnitude of the H, and the anomalous resistivity and thus the AHC equal zero. Therefore, the AHC in IrMn/YIG bilayers in Fig.2 in the text arises from the interaction between the IrMn and YIG layers and the noncollinear spin structure on the kagome lattice can be excluded in the explanations of the present galvanomagnetic results[1].

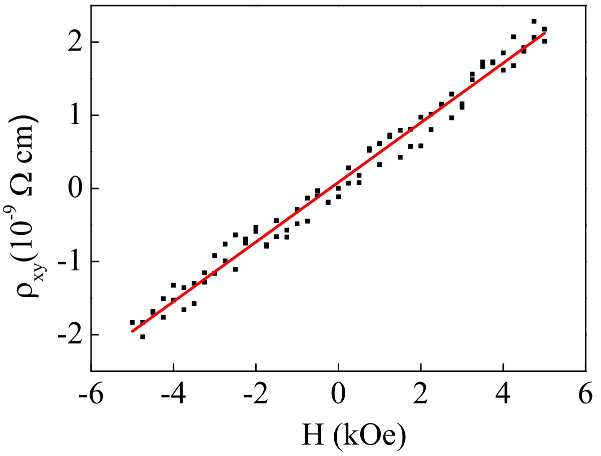

FIG. S2: For IrMn (5 nm) single layer film on the GGG substrate, Hall resistivity versus the H which is aligned along the film normal direction, where T = 10 K. The red line refers to a linear fit.

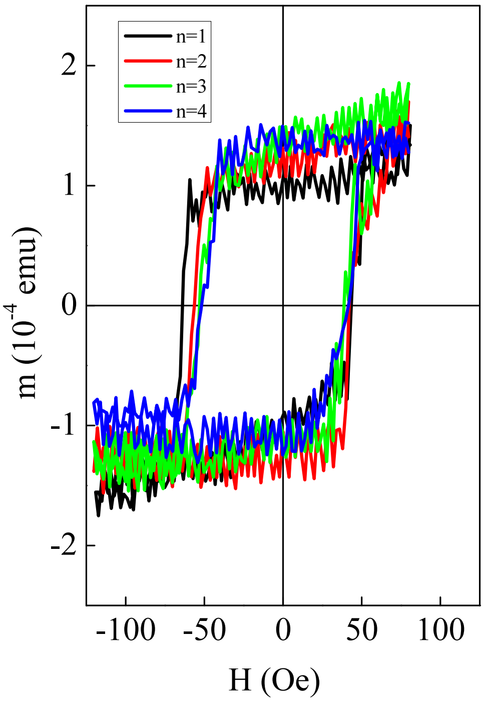

FIG. S3: For IrMn (5 nm)/YIG (20 nm) bilayers with an area of 0.25 cm^2, magnetization hysteresis loops with consecutive cycles, where $T = 40$ K.

D. Exchange bias training effect

In experiments, magnetic moments of the IrMn/YIG/GGG sample and the GGG substrate were first measured and the magnetic moment of the IrMn/YIG bilayer can then be obtained by subtracting the contribution of the substrate from the magnetic moment of the IrMn/YIG/GGG sample. Since the magnetic moment of the GGG substrate at low T is much larger than that of the IrMn/YIG bilayer, the magnetic noise is very large in the magnetization hysteresis loops. Since the noise becomes worse at low T, we can only show the magnetization hysteresis loops of the EB training effect at high T. Figure S3 shows that the magnetization loops with consecutive cycles. The exchange bias training effects measured by the planar Hall effect and magnetization loops are similar to each other and the reversal mechanism of the FM magnetization can be reflected by the ΔM_{AFM} in planar

Hall loops in Fig.3 in the text.

E. Temperature and angular dependent exchange bias

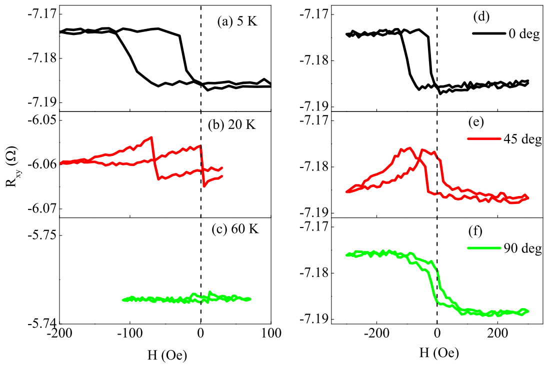

FIG. S4: For IrMn (5 nm)/YIG (20 nm) bilayers, planar Hall loops at $T = 5$ K (a), 20 K (b), 60 K (c) under the H along the sensing current, and at $\theta_H = 0$(d), 45(e), and 90(f) (degrees) at 5 K. θ_H refers to the angle between the H and the sensing current. The exchange field has a negative maximal value at $\theta_H = 0$ in (d), and it equals zero along the hard axis at $\theta_H = 90$ degrees in (f).

Figures S4(a)- S4(c) show the planar Hall loops at different T. The planar Hall loop is shifted from zero field at low T and centered $H = 0$ at $T = 60$ K. Meanwhile, the IrMn layer is far from saturation within the magnetic field of -200 Oe at $T = 5$ K and easily saturated within -100 Oe at high T. The observed T dependence agrees with the general variation trend of the exchange field and coercivity[2]. Moreover, the PHE signal decreases sharply with T. Therefore, the T dependence of the PHE signal is strongly related to the exchange bias. Planar Hall loops were also measured at different θ_H, as shown in Figs. S4(d)- S4(f). The angular dependent planar Hall loop reproduces the main feature of the angular dependence of the exchange bias that the exchange field and the coercivity decrease when the H deviates from the cooling field direction[3].

[1] H. Chen, Q. Niu, and A. H. MacDonald, Phys. Rev. Lett. **112**, 017205(2014)

[2] J. Nogués and Ivan K. Schuller, J. Magn. Magn. Mater. **192**, 203(1999)

[3] T. Ambrose, R. L. Sommer, and C. L. Chien, Phys. Rev. B **56**, 83(1997)
